# Mental health, study skills, social support, and barriers to seeking psychological help among university students: a call for mental health support in higher education

**DOI:** 10.3389/fpubh.2023.1220614

**Published:** 2023-10-18

**Authors:** Zamira Hyseni Duraku, Holly Davis, Era Hamiti

**Affiliations:** ^1^Department of Psychology, University of Prishtina “Hasan Prishtina”, Pristina, Kosovo; ^2^University Counseling Service, The University of Iowa, Iowa City, IA, United States

**Keywords:** youth, study skills, barriers to seeking psychological help, university mental health support, wellbeing

## Abstract

**Introduction:**

Poor mental health among youths is a complex worldwide issue. Many countries with medium-to-low levels of development, particularly those in Southern Europe, have not introduced appropriate mental health and educational strategies to identify the key factors influencing wellbeing, promote psychological wellbeing, and prevent poor mental health among youths. In response to these trends, we sought to uncover insights for developing interventions for youth mental wellbeing. We assessed mental health, study skills, barriers to seeking psychological help, and perceived social support among Kosovar university students, and investigated their experiences with professional mental health services and their needs and perceptions regarding the importance of professional mental health services on campus.

**Methods:**

The study used a parallel mixed-methods design. Participants included 234 university students. Quantitative data were gathered through validated questionnaires, including the Depression, Anxiety, and Stress Scale, Multidimensional Scale of Perceived Social Support, Academic Anxiety Scale, Study Skills Assessment Questionnaire, and the Barriers to Seeking Psychological Help Scale. Qualitative data on the students’ experiences with mental health services and their perceptions regarding the importance of professional university mental health services were explored through open-ended questions.

**Results:**

Most students experienced anxiety and depression, more than half were stressed, and most reported poor or moderate study skills. Lack of trust in mental health professionals was a major barrier to seeking psychological help, followed by difficulties in self-disclosure. Perceived social support and academic anxiety were significant predictors of barriers to seeking psychological help. The participants believed that mental health and academic support from the university would help improve their mental wellbeing, study skills, self-esteem, self-perception, and attitudes toward social support; raise awareness regarding mental health; and help them overcome personal and academic challenges.

**Discussion:**

Our findings highlight the need for more comprehensive and accessible mental health services on campuses. By providing adequate support and resources to address various personal and academic factors that contribute to mental health issues in university students, universities can enhance students’ academic success and personal growth.

## 1. Introduction

Mental health—which includes emotional, psychological, and social wellbeing—affects how people cope with stressful situations and make healthy decisions (1–3). Youths (aged 15–24 years) are at the highest risk of mental health problems, as half of all lifelong mental disorders start by age 14, and three-fourths by the mid-20s (4, 5). Mental health deterioration affects a youth’s skill development, social life, economic independence, and personal life, while also preventing them from successfully managing academic commitments (2, 5, 6).

Globally, one in seven (14%) young people experience mental health issues, although these symptoms remain largely undiagnosed and untreated (7). Multiple biological, social, and cultural factors influence a youth’s development, functioning, and mental health (5, 8). Typically, youths attend college or university between the ages of 18 and 24 years (9). Factors such as starting higher education, facing new challenges, making more decisions independently, and adapting to academic requirements and the new environment can affect their mental wellbeing (10, 11). Meanwhile, the inability to overcome these challenges can affect their mental health, which can in turn interrupt their studies (12) and limit academic achievement (6).

Poor youth mental health has become a worldwide issue, particularly in developing countries, where it is the most common and complex psychological problem (13). Additionally, mental health problems among youths have become especially notable in the wake of the coronavirus disease 2019 (COVID-19) pandemic. The first year of the pandemic saw an over 25% increase in anxiety disorders and depression among youths (5). Moreover, according to research with university students in 10 countries (the UK, US, Netherlands, France, Spain, Australia, and Nordic countries), 76% of participants struggled to maintain their wellbeing (14). During the 2020–2021 academic year alone, over 60% of participating students from 373 US campuses met the criteria for at least one psychiatric disorder (14, 15). Similarly, according to a study conducted in 12 European countries (including Kosovo), Asia, Latin and North America, and Australia, 48% of university students reported symptoms of depression (16).

Such an increase in the prevalence of mental disorders (particularly anxiety and depression) has negatively affected quality of life among youths, including academic performance, academic integrity, self-confidence, and interpersonal relationships; it even leads to suicidal thoughts (7, 17, 18). The youth suicide rate has increased in the past 60 years, and suicide is now the second leading cause of death in this demographic (19). Furthermore, while anxiety and depression often develop in comorbidity (20), these disorders also affect students’ learning abilities, such as completing academic tasks and attending classes (21). Hence, students’ learning skills—including memory, time management, test preparation, concentration level, and note-taking skills—not only affect their academic performance (22) but are also a protective factor against mental health deterioration or increased academic anxiety (23).

Several perspectives emphasize the need to identify key factors influencing youth wellbeing and notably offer insights for developing interventions to improve youth mental health. For example, the developmental systems approach (24), which draws from several developmental models, emphasizes the interaction between biological and environmental risks that can affect mental disorders. According to this perspective, interventions promoting mental health can prevent the further development of mental disorders if they are based on trends in competence and disorder rooted in age; different contexts; developmental activities; and biological, psychological, and social interactions (24, 25). Likewise, cooperation between family, community, and youths is necessary to develop and implement programs to protect mental health and ensure their sustainability. Moreover, these programs can become sustainable and efficient when services are extended in different dimensions, integrated into educational and health policies, and supported by local and national institutions (26).

The “Comprehensive Mental Health Action Plan 2013–2030” (27) specifies the importance of developing mental health services that consider health and social needs at all life stages, including adolescence and adulthood; it also clarifies the importance of developing multisectoral approaches. This plan provides guidance and suggestions for specific actions to be taken by all countries and parties involved in mental health. It aims to promote everyone’s mental health and wellbeing and prevent mental health deterioration among groups that are most at risk. Moreover, this plan recommends that mental health should be seen as an integral part of health and wellbeing.

Meanwhile, the task shifting approach is an alternative recommended for countries with limited resources. This model addresses challenges that mental health workers face in addressing multiple demands and helps improve mental health systems (28). Task shifting involves delegating the provision of support to certain groups; support is delegated from highly qualified professionals to those with fewer qualifications (29, 30). Additionally, the public health approach is rooted in the main principle of preventing problems before they develop. According to this perspective, to address mental health needs and adopt appropriate policies, it is important to identify factors that constitute the social, economic, physical, and geographic environments impacting the implementation of policies and intervention programs as well as citizens’ health (31).

Furthermore, the World Economic Forum and Orygen developed the “Youth Mental Health Framework” (32) to support countries with low-, medium-, and high-level resources in creating support/care systems and promoting the importance of mental health. These guides help implement mental health services that (1) are quick, easy, and accessible; (2) are focused on youth care; (3) bring attention and encourage comprehensive responses to existing issues; (4) are early-intervention programs; (5) foresee cooperation with youths; (6) have commitment and support from families; (7) involve continuous improvements; and (8) focus on prevention. According to this framework, youths should be supported holistically to reach their potential (32, 33).

The World Health Organization (27) also continuously calls for institutional and political representatives to do more for citizens’ mental health by bringing it out of the shadows and prioritizing it on a global scale. Moreover, researchers call for prioritizing the protection and promotion of youth wellbeing as highly as investments in other areas (e.g., economic, social, and educational) (34). While many countries have made progress along these lines, many others—especially middle-income countries—still devote less than 2% of their budgets to mental health; additionally, over 70% of mental health funding goes to treatments in psychiatric hospitals, which are usually associated with weak results (5, 27).

Higher education institutions are seen as ideal settings to promote and protect youth wellbeing by creating opportunities for students to reach their full potential (31, 32). In particular, the COVID-19 pandemic has prompted many universities to reflect on their practices and increase their commitment to student support; for example, some higher education institutions have been focusing on advancing mental health and academic programs that support students emotionally and academically. This trend has improved higher education institutions’ practices for supporting holistic student development (14). Additionally, students have been seeking more support from their universities because of anxieties related to finances and career development in light of the pandemic’s long-term impact (14). The US and Canada have achieved great advances in this area, including revising existing practices, examining opportunities, and improving student support to address the pandemic’s impact. However, less progress has been reported in European countries. In a recent study analyzing the challenges and needs of students and staff in universities across 11 European countries, including the UK, it was found that the mental health of both staff and students is a significant concern. The abovementioned study highlights the need for improved access to mental health services, enhanced university support, and promoting a culture of wellbeing (34). Moreover, some countries do not consider the wellbeing of their students a responsibility of higher education institutions but rather a private matter of students (35). Franzoi et al. (36) found that while most universities across Europe offer career counseling, many lack appropriate psychological support for their students. Furthermore, the primary approach to treating psychiatric disorders among these countries, particularly in the southern part of Europe, remains rooted in the medical model (5). However, other factors also affect youth mental health, including poor knowledge regarding the importance of mental health and stigma and discrimination against those seeking professional mental health services (5, 37, 38).

Kosovo is a post-conflict country in Southern Europe, bordered by Albania, North Macedonia, Montenegro, and Serbia. Youths constitute about 48% of the country’s population. Some risk factors that adversely influence the wellbeing, education, and economic independence of Kosovar citizens (especially youths) are as follows: economic difficulties; challenges in advancing education quality; and inadequate mental health services to address the war’s long-term psychological consequences, intergenerational trauma, and the impact of the COVID-19 pandemic (39–41). In Kosovo, the pandemic increased anxiety and stress among higher education students (39). Mental health stigma in Kosovo can prevent local youths from seeking the required help (37). Contextual factors, together with a lack of psychological services and social support, further marginalize specific groups of youths and increase their risk of poor mental health and underperformance while they complete their higher education. The World Bank Group (42) reported that 74% of the LGBTQIA+ community interviewed in Kosovo hesitated to express their sexual orientation owing to discrimination, violence, and non-acceptance. Moreover, when LGBTQIA+ youths declare their sexual orientation to others, they are at risk of losing family support and thus having to leave home (43). Similarly, the lack of social and psychological support and the inability of Kosovar educational institutions to create inclusive opportunities increases the risk of mental health problems for different groups of young people—including those who have chronic illness and disability, are pregnant or are parents, are from poor socioeconomical backgrounds, and are from ethnic minority groups (5, 44, 45).

The existing framework for educational and health policies in Kosovo may be further improved by including strategies for protecting and promoting youth psychological wellbeing. Currently, the Law on Kosovo Higher Education, which, among other things, aims to create a legal base for the quality assurance of Kosovar higher education in accordance with European standards, does not address the role of higher educational institutions in students’ wellbeing (46). The plan to achieve international quality standards is set out in the Education Strategy for 2022–2026, which was developed by the Ministry of Education, Science, Technology, and Innovation (47). These standards are used as criteria for the accreditation of study programs in Kosovo’s higher education institutions and measure performance to ensure external quality assurance (48). According to these standards, academic counseling, career planning, employment advice, and personal or psychological counseling services should be easily accessible to students throughout institutions (Standard 9.11) (48). However, the framework’s higher education strategy to improve services for students only emphasizes the capacity advancement of centers for career development (Item 4.3.4) (47). Notably, its four-year plan does not include psychological support services for students. Additionally, mental health services still need to be developed for young people in Kosovo’s primary and high schools; many public schools do not have counselors/psychologists (18). According to the Association of School Psychologists of Kosovo, the 1,058 educational institutions in Kosovo employ only 57 school psychologists/counselors and 50 pedagogues. Additionally, 50% of public high school students who participated in a recent survey conducted by the Kosovo Youth Council reported that they do not have psychologists at their school (49).

As for public mental health services, the total number of staff in mental health care in Kosovo is 160, while that in out-of-hospital care is 190—18.57 staff per 100,000 inhabitants (50). Counseling services are relatively new in the Kosovo system and are mainly offered by private practices, while mental health awareness programs for youths are usually organized by (1) non-government organizations as part of their specific projects or programs or (2) specific higher education institutions, through psycho-educational activities (18, 51). Further, the legal framework on mental health in Kosovo does not consider specific measures to promote citizens’ wellbeing. Although the Mental Health Law aims to protect and promote psychological wellbeing, it mainly focuses on the regulation of services for psychiatric disorders (Law Nr. 05/L-025) (52). Moreover, even the “Concept Document for Mental Health” to develop policies for preserving citizens’ wellbeing and health, recently published by the Ministry of Health in Kosovo (50), largely focuses on mental disorders, considering the treatment of people with mental health disorders the main problem.

Higher education in Kosovo is organized through public and private universities. The State of Kosovo has nine public universities and 14 private universities. According to 2020–2021 data, the major public higher education institution in Kosovo has 1,369 academic staff members with full-time employment and 722 external associates. According to the latest data, this university has approximately 30,450 students (53). The professor–student ratio in public institutions is 1:42. The combination of an insufficient number of academic staff and large groups of students is reported as a risk factor for the quality of higher education (54). Since the COVID-19 pandemic, the largest Kosovar public university has implemented some initiatives by specific faculty departments to offer psycho-education support for students so they can overcome challenges that impact their wellbeing and academic performance. However, these existing support programs are conducted voluntarily by students—that is, they are peer-to-peer support programs (supervised by professional mentors, university staff, and mental health workers). The services have not yet been integrated into the public university framework, mission, or strategies for higher education. As a result, their sustainability and the provision of more advanced professional services are at risk.

In this study, we aimed to assess the mental health, study skill level, barriers to seeking psychological help, and perceived social support among students at a major university in Kosovo. We also explored students’ experiences with professional mental health services, as well as their needs and perceptions regarding the importance of professional mental health support in higher education institutions. Most studies on issues related to higher education students’ mental health and wellbeing have been conducted in the US, Canada, Australia, and England; to date, there is a lack of research on this topic in other European countries as well as in Asia and Africa (55). Therefore, the evidence obtained from this study can serve as a starting point to accelerate the reformation and adaptation of legislative, educational, and social strategies for supporting youth psychological wellbeing. The findings may help countries with similar contextual characteristics address barriers and understand the state of students’ mental health and wellbeing in educational institutions. Furthermore, such scientific evidence can be vital for improving the quality of education and furthering educational institutions’ capacities to support their students’ emotional wellbeing and mental health (27, 56). These findings are particularly relevant for countries that have suffered conflict, including Kosovo, where advancing educational quality and reformation continues to be among the main challenges (57–59).

Moreover, the pandemic is expected to have long-term educational and economic impacts, further undermining young people’s mental health and future in both developed and developing countries. This will prevent students from realizing their full potential, including their career goals (5, 60). The lack of support services—in addition to other factors—continues to cause young people, such as students, to be at risk of declining mental health, which can threaten their academic and professional potential (5, 61). Therefore, the scientific evidence from this study can improve the advocacy necessary to quicken policy changes to support youth mental health—especially as new evidence-based strategies are considered most efficient and sustainable—and can serve as an important reference for improving the quality of education and commitments of university campuses (26, 27).

## 2. Materials and methods

### 2.1. Study design

We used a parallel mixed-methods design in which both quantitative and qualitative data were collected concurrently. In the quantitative part of the study, we examined university students’ mental health, study skills, barriers to seeking psychological help, and perceived social support. In the qualitative part, we explored their experiences and perceptions regarding the importance of professional university mental health services. The qualitative data helped provide context to complement the quantitative data. This comprehensive approach helped perform in-depth exploration of the needs for professional help and the contextual facilitators and barriers that students encounter when seeking psychological help in their academic and living contexts.

### 2.2. Study setting and participants

The study was conducted at a major university in Kosovo. Participants were undergraduate and graduate students from various study programs. Approval from the University’s Board of Ethics was obtained before conducting the study. Written informed consent was obtained from all participants, who were informed that their participation in the study was voluntary, their data would be kept confidential, and they could stop participating in the study at any time without any negative consequences.

### 2.3. Sampling technique and data collection

To ensure the sample’s diversity and representativeness, we targeted potential participants in the age range of 18–24, who are currently residing in either urban or rural areas across different regions of Kosovo. To be eligible, participants needed to be enrolled at the major university in Kosovo and be actively pursuing their studies at the undergraduate or graduate level. We sent potential participants a link to an online survey (kobotoolbox), using various methods (e.g., university email addresses and the student union network), and invited them to participate by completing the survey in their own time. To enhance participation, we sent the students two reminders to complete the online survey (with links). Students who did not complete the entire survey, were not enrolled at the above university, or did not fall within the age range of 18–24 were excluded from the study. Data were obtained from a total of 234 students—0.805% of the total student population in the sampling frame. The survey took approximately 45 min to complete. We gathered data from October to December 2022.

### 2.4. Materials

The survey included a series of validated questionnaires and open-ended questions. For the purpose of this study, the survey was translated and back translated into Albanian. To ensure the quality and suitability of the survey, a pilot study was conducted with 25 participants who met the inclusion criteria. These participants were asked to complete the survey and provide feedback on the wording, format, and length of the questions, as well as the ease and comfort of completing the survey. Based on the results of the pilot test, minor modifications were made to some of the questions to improve their clarity and relevance. Cronbach’s α was also used to test the internal consistency of all scales and subscales used in the survey.

#### 2.4.1. Multidimensional scale of perceived social support

Multidimensional scale of perceived social support (MPSS) is a 12-item instrument used to assess perceived social support. It comprises 12 items divided into three subscales of four statements each associated with the social support source, namely, family, friends, and significant others. Participants are asked to rate each statement on a 7-point Likert scale (1 = *very strongly disagree* to 7 = *very strongly agree*). To calculate each subscale’s mean scores, subscale items are summed and divided by 4. For the total scale’s overall mean score, the sum of all 12 items is divided by 12 (62). The overall scale’s internal consistency for the current study was excellent (α = 0.91).

#### 2.4.2. Academic anxiety scale

Academic anxiety scale (AAS) is a psychometric tool used to measure academic anxiety. This scale includes 11 items that evaluate perceived stressors contributing to academic anxiety among participants, such as their reactions to academic tasks and their worries, fears, and confidence. All items are scored on a 4-point rating scale (1 = *not at all typical of me* to 4 = *very typical of me*); the overall score represents the response values’ total (63). The overall scale’s internal consistency for the current study was excellent *α* = 0.91.

#### 2.4.3. Depression, anxiety, and stress scale

Depression, anxiety, and stress scale (DASS21) measures depression, anxiety, and stress levels. The 21 items are divided into three self-report scales with seven items each. Each scale’s scores are determined by summing the scores for the relevant items and multiplying them by 2. The cutoff scores for severity are as follows: Depression is considered normal at 0–9, mild at 10–13, moderate at 14–20, severe at 21–27, and extremely severe at ≥28. Anxiety is scored as normal at 0–7, mild at 8–9, moderate at 10–14, severe at 15–19, and extremely severe at ≥20. Stress is scored as normal at 0–14, mild at 15–18, moderate at 19–25, severe at 26–33, and extremely severe at ≥34 (64). For the current sample, the internal consistencies were as follows: depression, excellent (*α* = 0.92); anxiety, excellent (*α* = 0.93); stress, good (*α* = 0.89); and the overall scale, excellent (*α* = 0.94).

#### 2.4.4. Study skills assessment questionnaire

The Study Skills Assessment Questionnaire evaluates participants’ study skills across eight sections, each containing eight items answered on a 4-point rating scale (1 = *never* to 4 = *always*). These sections measure different aspects of study skills. Total scores for each section indicate the participants’ level of study skills (<20, poor; 21–28, moderate; >28, good). For the overall scale, lower scores indicate poorer study skills (65). The subscales’ internal consistencies for the current sample were as follows: time management and procrastination, good (*α* = 0.84); concentration and memory, acceptable (*α* = 0.74); study aids and note-taking, good (*α* = 0.85); test strategies and academic anxiety, acceptable (*α* = 0.75); organization and processing of information, good (*α* = 0.84); motivation and attitude, good (*α* = 0.81); reading and selection of the main idea, good (*α* = 0.82); and writing, acceptable (*α* = 0.73). The overall scale’s internal consistency was excellent at *α* = 0.95.

#### 2.4.5. Barriers to seeking psychological help scale

This instrument is used to determine the various obstacles to seeking psychological help, such as fear of societal stigma, lack of trust in mental health professionals, difficulties in self-disclosure, perceived devaluation, and lack of knowledge (66). This 17-item self-report scale has five subscales. Participants indicate how much they agree with each item on a 5-point Likert scale (1 = *strongly disagree* to 5 = *strongly agree*). Total scores for the fear of societal stigma and lack of trust in mental health professionals subscales range from 4 to 20, whereas those for other subscales—difficulties in self-disclosure, perceived devaluation, and lack of knowledge—range from 3 to 15. Higher scores indicate greater obstacles regarding the respective subscale and overall scale. The subscales’ internal consistencies for the current sample were as follows: fear of being stigmatized by society, good (*α* = 0.85); trust in mental health professionals, good (*α* = 0.84); difficulties in self-disclosure, acceptable (*α* = 0.75); perceived devaluation, acceptable (*α* = 0.77); and lack of knowledge, good (*α* = 0.84). The overall internal consistency of the scale was excellent at *α* = 0.93.

#### 2.4.6. Contextual facilitators and barriers to accessing mental health services

The survey included a series of open-ended questions asking participants to describe their previous experience (if any) with mental health services, articulate reasons for not utilizing mental health support services despite recognizing the need, and provide insights into the availability of mental health services within their university context. Further, participants were asked to express their opinions regarding the importance of mental health services at the university, and also identify areas in which mental health support is vital for students. The responses to these open-ended questions constituted the qualitative dimension of our study.

#### 2.4.7. Demographics

Information regarding participants’ sociodemographic characteristics—including age, gender, sexual orientation, educational level, ethnicity, employment status, civil status, living arrangement, urbanicity, parental educational level, and socioeconomic status—was collected in the first section of the survey.

### 2.5. Data analysis

#### 2.5.1. Quantitative analysis

A summary analysis was performed for each categorical variable using frequencies and percentages; for continuous variables, means (SDs) and medians (interquartile range) were used. Normality tests were performed using Kolmogorov–Smirnov and Shapiro–Wilk test. Spearman’s correlations were used to examine the relationships between the scale scores of different groups as they were found to have a non-normal distribution. A chi-square test helped determine whether there were any differences between the scores of different dichotomous groups. Logistic regression was conducted to predict the different factors contributing to the barriers to seeking psychological help after adjusting for potential confounders. For the closed-ended questions, statistical analyses were conducted using SPSS version 26 (IBM Corp., Armonk, NY, USA).

#### 2.5.2. Qualitative analysis

Participants’ responses to the open-ended questions were subjected to a deductive thematic content analysis, encompassing the identification, analysis, categorization, and reporting of patterns (themes) inherent within the data. Guided by the research objectives and subject matter, the qualitative investigation revealed the following four predetermined distinct themes: (1) Participants’ previous experiences with professional mental health services; (2) Barriers to utilizing mental health services; (3) Perceived need for mental health services at the university; and (4) Areas for mental health service support.

Considering the diverse language backgrounds of the researchers, the qualitative data, which were originally gathered in the Albanian language, were translated into English to facilitate collaboration and accurate data analysis. Furthermore, to ensure fidelity of the translated content, a back-translation process was also employed, in which the English version of the content was translated into the original (Albanian) language to verify consistency and accuracy. The data analysis and interpretation were based on the English-translated transcripts to maintain linguistic consistency within the research team and for the final report.

To ensure the rigor and dependability of the qualitative analysis, a two-fold process was employed. First, the data were analyzed independently by the authors, who collaborated to align the aspects to be investigated. Prior to report writing, a subset of the data was reviewed, and the percentage of agreement was calculated to validate the consistency of the interpretations. The inter-rated reliability compliance rate was 91.0%. The coding software NVivo 14 (2023) was used to categorize the frequency of responses and generate the corresponding codes. The thematic analysis was facilitated by using ATLAS.ti 22.

## 3. Results

Table 1 shows all participants’ demographic characteristics. The majority (95.3%) of the participants were undergraduate students. Their mean age was 19.29 years (standard deviation [*SD*] *=* 1.69).

**Table 1:** Participants’ demographic characteristics (*N* = 234).

Characteristics	*N*	%
Gender		
Female	202	86.3
Male	29	12.4
Prefer not to answer	3	1.3
Sexual Orientation		
Heterosexual	184	79.0
LGBT	16	14.0
Prefer not to answer	34	7.0
Level of Study		
Undergraduate: BA	223	95.3
Graduate: MA	11	4.7
Ethnicity		
Albanian	230	98.0
Turkish	4	2.0
Employment status		
Not employed	203	86.8
Employed	31	13.2
Civil Status		
Single	191	82.0
In a relationship	33	14.0
Engaged	7	3.0
Married	1	1.0
Living arrangement		
Lives with family	120	51.0
Lives on campus/with roommates	111	48.0
Lives alone	3	1.0
Urbanicity		
Rural	97	41.4
Urban	137	58.6
Parents completed higher education		
No	149	64.0
Yes	85	36.0
Chronic disease and disability		
No	230	98.0
Yes	4	2.0
Socioeconomic status		
Low	18	8.0
Lower- Middle	163	70.0
Upper-Middle	38	16.0
High	15	6.0
	Mean	SD
Age	19.29	1.69

BA: Bachelor of Arts, MA: Master of Arts; LGBT: lesbian, gay, bisexual, and transgender; Chronic disease and disability: attention-deficit/hyperactivity disorder, depression, generalized anxiety disorder, stuttering.

The results show that most participants reported symptoms of anxiety and depression—190 reported being extremely anxious, 152 reported signs of severe depression, and more than half (*n* = 134) were stressed (see Table 2).

**Table 2:** Prevalence of depression, anxiety, and stress among the study participants (*N* = 234).

Level	Depression *n* (%)	Anxiety *n* (%)	Stress *n* (%)
Normal	22 (9.4)	16 (6.8)	100 (42.7)
Mild	4 (1.7)	6 (2.6)	30 (12.8)
Moderate	28 (12.0)	13 (5.6)	39 (16.7)
Severe	28 (12.0)	9 (3.8)	39 (16.7)
Extremely severe	152 (64.9)	190 (81.2)	26 (11.1)

Further, most students reported having poor or moderate study skills and reported needing improvements in all measured areas. For example, 56.0% reported lacking time management skills, only 59.0% reported having moderate skills in testing strategies and academic anxiety, and only 57.3% reported having moderate reading skills (Table 3).

**Table 3:** Study skills levels among the study participants (*N* = 234).

Level	Time management	Concentration	Note-taking	Test strategies and academic anxiety	Organization and processing of information	Motivation	Reading	Writing
	*n* (%)	*n* (%)	*n* (%)	*n* (%)	*n* (%)	*n* (%)	*n* (%)	*n* (%)
≤ 20 (poor)	131 (56.0%)	88 (37.6%)	71 (30.3%)	78 (33.3%)	69 (29.5%)	92 (39.3%)	62 (26.5%)	88 (37.6%)
21–28 (Moderate)	87 (37.2)	124 (53.0%)	124 (53.0%)	138 (59.0%)	127 (54.3%)	118 (50.4%)	134 (57.3%)	125 (53.4%)
> 28 (Good)	16 (6.8%)	22 (9.4%)	39 (16.7%)	18 (7.7%)	38 (16.2%)	24 (10.3%)	38 (16.2%)	21 (9.0%)

Table 4 presents the results of an item-wise analysis of academic anxiety. The results indicated that 36.3% of students did not get a good night’s rest before an exam. Furthermore, 15.4% of students never felt confident about their level of preparedness for exams, and 13.7% reported an inability to calmly recall what they know during an exam.

**Table 4:** Item-wise analysis of academic anxiety among the study participants (*N* = 234).

Statement	Never *n* (%)	Sometimes *n* (%)	Usually *n* (%)	Always *n* (%)
I try to find out what the exam will cover and how the exam is to be graded.	11 (4.7%)	40 (17.1%)	78 (33.3%)	105 (44.9)
I feel confident that I am prepared for the exam.	36 (15.4%)	109 (46.6%)	59 (25.2%)	30 (12.8%)
I try to imagine possible test questions during my preparation for an exam.	8 (3.4%)	40 (17.1%)	90 (38.5%)	96 (41.0%)
I take time to understand the exam questions before starting to answer.	8 (3.4%)	51 (21.8%)	95 (40.6%)	80 (34.2%)
I follow directions carefully when taking an exam.	7 (3.0%)	23 (9.8%)	84 (35.9%)	120 (51.3%)
I usually get a good night’s rest prior to a scheduled exam.	85 (36.3%)	67 (28.6%)	50 (21.4%)	32 (13.7%)
I am calmly able to recall what I know during an exam.	32 (13.7%)	86 (36.8%)	71 (30.3%)	45 (19.2%)
I understand the structure of different types of tests and am able to prepare for each type.	22 (9.4%)	84 (35.9%)	74 (31.6%)	54 (23.1%)

Trust in mental health professionals (*M* = 9.58, *SD* = 4.43) was a major factor contributing to the barriers to seeking psychological help, followed by difficulties in self-disclosure (*M* = 8.00, *SD* = 3.40), fear of being stigmatized by society (*M* = 7.72, *SD* = 3.94), perceived devaluation (*M* = 6.54, *SD* = 3.14), and lack of knowledge (*M* = 6.27, *SD* = 2.71), showing a significant difference between the different factors contributing to barriers to seeking professional help (*p* < 0.001).

Regarding perceived social support, the participants perceived similar support from different sources—significant other (*M* = 4.77, *SD* = 1.49), family (*M* = 4.77, *SD* = 1.34), and friends (*M* = 4.69, *SD* = 1.40) —indicating no significant difference between the perceived social support from all sources (*p* = 0.759).

Spearman’s correlations were used to examine the relationships between the scores of different scales and ordinal groups (Table 5). A chi-square test was conducted to determine if there were any differences between the scores of different dichotomous groups. Significant differences were observed between age and study skills (rho = 0.157, *p* = 0.016), suggesting that older participants had significantly better study skills than younger participants. Female participants exhibited significantly higher scores on depression, anxiety, and stress (*p* = 0.002). Furthermore, significant differences were noticed among female participants and perceived social support (*p* = 0.027). Notably, sexual orientation was significantly associated with academic anxiety (*p* = 0.035), particularly among participants with LGBT identification. Moreover, a lower family socioeconomic status was significantly associated with barriers to seeking professional help (*p = 0.037*) and perceived social support (*p* = 0.018).

**Table 5:** Bivariate analysis of depression, anxiety, and stress; academic anxiety; study skills; barriers to seeking professional help; and perceived social support with socio-demographic characteristics among the study participants.

Factors	Depression, anxiety, and stress	Academic anxiety	Study skills	Barriers to seeking professional help	Perceived social support
Age	rho = 0.015 (*p* = 0.234)	rho = −0.053 (*p* = 0.234)	rho = 0.157 (*p* = 0.016) *	rho = −0.101 (*p* = 0.124)	rho = 0.094 (*p* = 0.234)
Gender	χ^2^ = 12.516 (*p* = 0.002) *	χ^2^ = 3.761 (*p* = 0.153)	χ^2^ = 4.990 (*p* = 0.083)	χ^2^ = 2.184 (*p* = 0.335)	χ^2^ = 7.250 (*p* = 0.027) *
Sexual Orientation	χ^2^ = 29.496 (*p* = 0.091)	χ^2^ = 12.013 (*p* = 0.035) *	χ^2^ = 5.458 (*p* = 0.363)	χ^2^ = 9.759 (*p* = 0.082)	χ^2^ = 2.166 (*p* = 0.826)
Level of Study	χ^2^ = 1.215 (*p* = 0.270)	χ^2^ = 0.263 (*p* = 0.608)	χ^2^ = 7.890 (*p* = 0.005) *	χ^2^ = 2.476 (*p* = 0.116)	χ^2^ = 0.362 (*p* = 0.548)
Socioeconomic status	χ^2^ = 4.538 (*p* = 0.338)	χ^2^ = 6.431 (*p* = 0.169)	χ^2^ = 4.006 (*p* = 0.405)	χ^2^ = 10.234 (p = 0.037) *	χ^2^ = 11.900 (p = 0.018) *
Parental educational level	χ^2^ = 2.098 (*p* = 0.148)	χ^2^ = 2.632 (*p* = 0.105)	χ^2^ = 1.748 (*p* = 0.186)	χ^2^ = 3.764 (*p* = 0.052)	χ^2^ = 0.612 (*p* = 0.434)
Chronic disease and disability	χ^2^ = 1.240 (*p* = 0.265)	χ^2^ = 1.280 (*p* = 0.258)	χ^2^ = 0.983 (*p* = 0.321)	χ^2^ = 0.001 (*p* = 0.986)	χ^2^ = 0.704 (*p* = 0.401)

*p < 0.05.

The logistic regression model was developed to predict barriers to seeking psychological help after adjusting for confounding factors (Table 6). Perceived social support (*p* = 0.002) and academic anxiety (*p* = 0.001) were significant predictors of barriers to seeking psychological help; meanwhile, depression, anxiety, and stress (*p* = 0.139) and study skills (*p* = 0.153) were not.

**Table 6:** Regression model: predictors of barriers to seeking professional help among the study participants.

Variables	Coefficient (*B*)	*SE (B*)	Value of *p*	Odds ratio Exp(B)	95% CI	
					Lower bound	Upper bound
(Constant)	22.328	19543.943	0.999			
Socioeconomic status			0.348			
Low	−23.327	19453.943	0.999	0.000	0.000	–
Lower- middle	−21.976	19453.943	0.999	0.000	0.000	–
Upper-middle	−21.978	19453.943	0.999	0.000	0.000	–
High	−22.793	19453.943	0.999	0.000	0.000	–
Depression, anxiety, and stress	−0.525	0.355	0.139	0.592	0.295	1.186
Perceived social support	−0.999	0.319	0.002*	2.716	1.453	5.079
Academic anxiety	−1.451	0.356	0.001*	0.234	0.117	0.471
Study skills	0.442	0.309	0.153	1.556	0.849	2.853

SE, standard error; CI, confidence interval; *significant value of *p*.

Below, we present the results along with some of the participants’ answers to the open-ended questions based on the four themes described in the methodology section.

### 3.1. Theme 1: previous experience with professional mental health services

In total, 14.01% participants reported having previous experience with professional mental health services, including psychotherapy, school counselor support, and psychiatrist and neurologist visits. Students found psychotherapy and school counseling support to be beneficial, while they expressed dissatisfaction with other approaches, such as medical interventions.

Some notable participant quotes are as follows:

“I went to a psychiatrist, who prescribed medication to me. I stopped visiting and taking the medication after two weeks. I decided to help myself by exercising and eating healthy.” – Male graduate student, 21 years old.

“I was diagnosed with [XX] and had problems with my self-esteem. I attended a few psychotherapy sessions, which were very helpful in improving my self-esteem and social life.”– Male undergraduate student, 19 years old.

These narratives offer insights into the diverse experiences of participants with mental health services, complementing the quantitative data by providing a nuanced understanding of student’s attitudes toward different types of mental health services.

### 3.2. Theme 2: barriers to utilizing mental health services

Most students (86%) had never used any type of mental health support services, despite believing that they needed such support. The reasons for not using mental health services included the lack of services; stigma experienced by self, family, and friends toward using mental health services; difficulty in sharing personal issues with others; skepticism regarding the ethical principles (confidentiality and professional competence) of mental health workers in their country; and inability to afford the services.

Some notable participant quotes are as follows:

“I believe that mental health support services are very important; yet, there are no such services, either within our schools or where we live.”– Female undergraduate student, 18 years old

“Stigma regarding mental health—starting with myself, my family, and my friends—has prevented me from seeing a psychologist.” – Male graduate student, 20 years old

“I have doubts about the professional competency and integrity of mental health professionals in Kosovo.” – Female undergraduate student, 19 years old

“…at this stage of my life, I cannot afford to pay for counseling or any mental health services.” – Female undergraduate student, 18 years old

These qualitative insights align with the quantitative findings; strengthen the understanding of the barriers faced by students when seeking psychological help, such as distrust in mental health professionals, difficulties in self-disclosure, and fear of societal stigma. Additionally, these narratives illuminate other contextual barriers to accessing mental health services, including the lack of appropriate and accessible mental health support and financial constraints.

### 3.3. Theme 3: perceived need for mental health services at the university

A consensus emerged among most participants (95%) regarding the importance and necessity of mental health services at the university, as currently, such services are not available to them. The different forms of psychosocial activities mentioned as helpful included individual and group counseling, psycho-educational workshops and lectures, mental health literacy activities, and support groups. They also expressed the desire for professors to play a more active role in understanding their individual learning needs and protecting their wellbeing.

Some notable participant quotes are as follows:

“Most students don’t talk about their issues or personal concerns with their family or friends. Psychological counseling services, like individual or group counseling, would help us address these issues.” – Female undergraduate student, 19 years old

“We need professional psychological support at the university. Despite supporting us in overcoming stress, such services—if they include psycho-educational activities—would also help us raise awareness about the importance of wellbeing, which according to me is still very low.” – Male undergraduate student, 19 years old

“I believe that apart from being provided with psychological and emotional support, it is also very important for us to receive significant support from our professors. The professors should engage with us more, understand our individual educational needs, and motivate us to excel in our studies.”

– Female graduate student, 20 years old

“The right and a more supportive approach toward us from our professors would help us have better study skills and academic performance.” – Male undergraduate student, 19 years old

This insight highlights the need for mental health support at the university. Participants emphasized the importance of a holistic approach combining psychological support with academic guidance and its potential positive impact on their wellbeing and academic performance. Additionally, these narratives highlight the necessity of enhancing students’ study skills, a need also indicated by quantitative findings. Furthermore, the call for increased mental health awareness, as mentioned in the above narratives, also aligns with the quantitative findings, where a lack of knowledge has emerged as a significant barrier hindering students from seeking psychological help.

### 3.4. Theme 4: areas for mental health service support

The participants identified several areas in which mental health services could positively impact their lives. These included sustaining and improving their mental wellbeing, enhancing study skills, raising awareness regarding mental health, overcoming their personal and academic challenges, improving their self-esteem and self-perception, becoming more open-minded and satisfied, and improving their attitudes toward social support. They also emphasized that such support would make them feel more supported by the university.

Some notable participant quotes are as follows:

“Students would be able to establish a healthy routine and pay more attention to their mental health.” – Male graduate student, 22 years old

“Students would become more motivated to learn, and their self-confidence would increase.” – Female undergraduate student, 19 years old

“We would be able to overcome our academic challenges and tackle the high levels of stress and anxiety.” – Male graduate student, 21 years old

“The university should support students’ mental health, especially during exams, when mental health is very poor.” – Female undergraduate student, 18 years old

These narratives illuminate the need for addressing students’ mental wellbeing, including high levels of stress and academic anxiety, in alignment with the quantitative findings. Furthermore, participants stressed that university mental health services could also aid in improving their study skills and overcoming personal and academic challenges, ultimately contributing to the development of more confident and motivated learners.

## 4. Discussion

We observed that Kosovar university students reported high rates of anxiety, depression, and stress, similar to the findings of other studies (17, 67). Moreover, the lack of mental health services within Kosovo and its education system, as reported in this study and previous research (18, 49), indicate that this population has a greater risk of mental health decline and being unable to achieve their full potential in higher education, manage their personal and social lives, and benefit from future employment opportunities (2, 5, 6, 67).

Further, the current findings demonstrated that the students generally perceived different sources, including family, friends, and significant others, as offering similar support. Additionally, social support predicted the barriers to youths seeking psychological help. However, it is important to note that our results also suggest potential disparities in perceived social support across different youth groups, such as females and those from lower socioeconomic backgrounds. Female participants, as noted in our results, showed higher significant scores on stress, anxiety, and depression. Therefore, these findings complement those of previous studies suggesting that socio-contextual and economic factors can form additional barriers for specific groups of youths seeking support (5, 18, 42–45). The above findings also underscore the importance of investigating specific stressors, especially those arising from contextual and cultural barriers, which can influence youth wellbeing and their level of social support (68). Identifying these factors can contribute to the development of effective interventions to mitigate mental health stigma across various sociodemographic youth groups and contribute toward safe and supportive environments for protecting mental health and supporting wellbeing (69).

Furthermore, poor study skills, academic anxiety, and insufficient support from educational staff and professors in addressing the participants’ academic obstacles were identified as risk factors that can negatively impact students’ academic achievement. Academic anxiety also emerged as significant predictor of barriers to seeking psychological help, offering a deeper understanding of students’ challenge. All these represent the challenges of Kosovo’s education system. Moreover, previous research has found that Kosovo’s public universities have an insufficient ratio of academic staff members/professors per student (1:42 ratio) (54). Furthermore, the results of the current study highlight that specific groups of students are more prone to academic anxiety. Students from the LGBT community showed significantly higher scores for test anxiety than students not part of the community. This result calls for creating an academic environment that embraces inclusivity and diversity and addresses the unique challenges students might face. Furthermore, our study’s findings, similar to those of previous studies, suggest that younger students might require additional support in advancing their study skills, a need that might stem from the transition to higher education with its associated challenged and adjustments facing new challenges and trying to adjust to higher education expectations (10, 11).

In this study, the barriers participants experienced in seeking psychological help included, among others, trust in mental health workers, hesitancy to seek psychological help, and stigma toward mental health issues where they live. Notably, the study population’s experiences with mental health services were relatively infrequent, and no other data have yet been collected on the quality of existing mental health services in Kosovo. The relative scarcity of existing work on the topic might be owing to the lack of adequate opportunities, programs, and possibilities for decreasing the stigma toward seeking professional help; for helping youths overcome challenges related to personal life and education; and for increased awareness of the roles of professional mental health workers and their ethical principles. The lack of accessibility to mental health services in the university was also raised as an issue by study participants. Previous studies have indicated that the development of mental health services would help improve the national quality of such services, citizens’ mental health, and quality of education, as well as reduce the stigma toward seeking psychological help (18, 70, 71). Furthermore, services that are appropriate to the needs of the youth and affordable can also help decrease barriers toward seeking psychological help among different groups of youth. This resonates with our study findings that youth from lower socioeconomic backgrounds encounter higher barriers in seeking psychological help. Therefore, establishing affordable mental health services tailored to the needs of the youth can help address these disparities (72).

### 4.1. Practical implications

Kosovo and other similar countries—that is, countries that mainly focus on treating mental health disorders in medical (psychiatric) settings and lack national strategies for prevention—should consider multisectoral approaches. These include (1) improving inter-institutional cooperation for concerns such as health, education, and social welfare and (2) enhancing cooperation among families, communities, and young people to develop and implement sustainable programs that promote and protect youth mental health, as indicated in the developmental systems approach and suggested by the Comprehensive Mental Health Action Plan (26, 27). Such prevention programs are more economical and have multiple positive benefits beyond the health sector in addition to protecting the citizens’ wellbeing. They can also improve future employment opportunities and education quality in the respective countries (27, 31, 61). Furthermore, similar to the public health approach, preventing problems before they could emerge, measuring multiple mental health risk factors, and taking appropriate measures to prevent and intervene should also be considered while further adapting and reforming public health and education strategies (31).

The other approach worth considering, owing to the lack of resources, would be task shifting. By increasing the cooperation among sectors and delegating the provision of support from psychiatrists to other professional mental health workers (e.g., psychologists and social workers), the mental health system can be improved, and youths can gain more opportunities for support (29, 30). Furthermore, higher education institutions should integrate psychological, academic, and social support services for students on campus (73, 74). These support programs can promote the importance of mental health and prevent mental health deterioration. However, they should be developed according to the mental, educational, and social needs of students and enable them to more easily overcome challenges related to education, improve the quality of education, and reduce the stigma toward seeking psychological help (70, 71).

In addition, integrating mental health services within education institutions may be the best way to reduce the social and economic costs to the country related to mental disorders (61). Thus, ensuring that the promotion of positive mental health and advancement of knowledge about mental health among all parties and beneficiaries of higher education is an integrated part of educational policies and curricula is important. Psychological support services can be developed as special counseling programs to protect students’ wellbeing. These can be offered in the universities’ counseling centers. As these services are an integrated part of the higher education mission and support it in different ways, it is important to maintain their efficiency. Some examples of the services include individual and group counseling, outreach (prevention and education), and training (mentoring and development of peer support and mental health professionals) (75, 76).

Such services have existed for more than 80 years in the US (74). In the last 40 years, their number has increased dramatically worldwide, and the variety of services offered has also increased (75, 77). Such counseling centers are integrated into several universities in European Union countries, such as Belgium, Croatia, Estonia, Finland, France, Germany, Hungary, Iceland, Ireland, Italy, Latvia, Lithuania, Luxembourg, Malta, Norway, Poland, Republic of Cyprus, Slovakia, Slovenia, Spain, Sweden, and the Netherlands (78), as well as Turkey (79).

Although counseling centers may differ in how they operate and the types of support programs they offer, their general purpose is to provide clinical mental health services, support students in overcoming academic challenges, advance their personal wellbeing and academic skills, prevent declines in mental health, and refer students to other needed professional services according to existing resources in the respective countries. Many of these centers also serve as opportunities for professional development for students in the field of psychology and counseling (75). Though each country might have different requirements and specifications, the quality of these centers is usually measured through different components: as developing their programs to meet the accreditation criteria specified in the standards of the International Organization for the Accreditation of Professional Counseling Centers in Higher Education Academic Campuses; having internal quality assurance processes; having professional and competent staff; and working in compliance with the country’s legal and ethical requirements (80). These centers serve as separate entities under the umbrella of “student affairs or wellness.” Therefore, although counselors engaged in counseling centers may also act as academic staff, the centers employ mental health professionals with a doctorate in psychology or master’s in counseling psychology/social work (e.g., psychologists, social workers, and counselors). The centers should have specific protocols to address students’ mental and emotional issues, measure the quality of their services as well as contribute toward their staff’s professional development (81).

Numerous studies testify to the efficiency of these services in addressing various emotional and academic aspects of student life, most of which relate to the needs of the current study population as well. Academic support, especially for students with learning difficulties, helps improve mental health (82). Psycho-educational activities are effective in reducing stress, anxiety, and general distress in students (83). Relaxation activities, which are also part of certain programs within these counseling centers, can efficiently protect student wellbeing (82). Peer support groups also have multiple positive effects on mental wellbeing and academic engagement while enabling students to support each other by sharing knowledge, offering emotional support, creating opportunities for social interactions, providing assistance, advocating, and increasing awareness (5, 84).

With the rapid development of technology in recent years, most services for students have been digitized. These include mental health services, which are provided online in some countries. Therefore, digital mental health services should be considered an additional option for supporting youth mental health. These services include support through web-based platforms, also called eHealth and mHealth interventions. A review (85) shows that symptoms of stress, anxiety, depression, and eating disorders might be reduced through web-based online interventions. Among the digitally delivered services, those that use one of the following six types of facilitation have the greatest effect: web-based services, digital versions of cognitive behavioral therapy, short messaging services and smartphone applications, interventions using virtual reality, relaxation and skills training, and exposure-based therapy (85). Digital services have also been effective in reducing the stigma associated with mental illness (86).

One of the needs declared by the participants in the current study was for additional support from their professors. Therefore, university staff should continuously engage in professional development to support students’ individual needs and advance their pedagogical and working approaches. This will stimulate learning and protect students’ mental health. Furthermore, in Kosovo and similar countries, measuring the quality of current mental health services, strengthening the legal base regulating mental health workers/practitioners’ professional competencies, and improving the professional abilities of mental health workers, especially their ethical standards, is critical to increase public mental health support, including support designed specifically for students, and strengthen public trust in psychological support. Public awareness regarding mental health professionals’ training and competencies, as well as the ethical standards that should be expected, would also help raise awareness and decrease stigma toward seeking psychological help.

### 4.2. Limitations and future directions

This study’s results provide a reference for assessing youths’ needs and promote the advancement of their mental health and educational support, especially in higher education. Nevertheless, this study has several limitations. First, due to the use of convenience sampling and the limited sample size, the results cannot be generalized to all youths or students at the major university in Kosovo. Second, the sample was limited in terms of disproportionateness in levels of education (undergraduate and graduate), programs of study, faculties, sexual orientation, and representative gender ratios. Consequently, the current study’s comparison findings with the aforementioned differences in student characteristics and demographics should be interpreted with caution. Therefore, these additional characteristics should be considered in the future to ensure a better and deeper understanding of higher education students’ needs for further support from the university. Third, it’s important to note that while perceived social support and academic anxiety emerged as significant predictors of barriers to seeking psychological help, the current study did not find a significant association between depression, stress, and anxiety, as well as study skills, and barriers to seeking psychological help. This lack of significance might be attributed to various factors, including the complexity of how these variables interact or influence help-seeking behaviors. Future research should further explore the interactive relationship between these variables and barriers to seeking psychological help. This exploration could also encompass more in-depth analyses that further assess the coping mechanisms students’ employ to manage their mental wellbeing by also considering cultural context’s influence on barriers to seeking psychological help. Furthermore, the relatively long data collection period (October to December 2022), which covered different periods of the students’ academic journeys over one semester (e.g., exams) as well as other external stressors that might have impacted their wellbeing (e.g., holidays) might have influenced the results. Future studies conducted over shorter periods or in multiple waves, as well as those that use longitudinal designs to address the influence of varying academic periods and external stressors on students’ wellbeing, can contribute to a more comprehensive understanding of students’ needs and offer interventions to support their mental health, individual needs, and study skills. Investigating other personal and academic factors that influence the above would also be helpful for all actors in the education and public health sectors: policymakers, administrators in educational institutions, staff members, and youths.

## 5. Conclusion

The study provides a comprehensive view of the mental wellbeing challenges faced by Kosovar university students. The high rates of anxiety, depression, and stress found in this study are consistent with that found in similar research. The lack of accessible mental health services within the country as well as in the education system suggests that university students are exposed to a heightened risk of developing mental health issues, hampering their academic and personal growth. The findings emphasize the significance of academic anxiety and social support, both of which appeared to predict barriers to seeking psychological help. The barriers to seeking psychological help, stemming from mistrust, stigma, and limited access, reflect the broader challenges in mental health provision. Thus, strong mental health services within the education system are essential to enhance overall wellbeing, decrease the stigma surrounding mental health, boost academic success, and promote the holistic development of the students. In sum, this study’s findings are relevant to advance knowledge of mental health’s importance among youths and thus advocate for greater, more appropriate psychological support. This will ensure the advancement of professional services in all educational institutions and help young people become active in decision-making processes related to their life and education (87).

## Data availability statement

The raw data supporting the conclusions of this article will be made available by the authors, without undue reservation.

## Ethics statement

Research approval from the University of Prishtina, Faculty of Philosophy and informed written consent from all participants were obtained prior to conducting the study.

## Author contributions

ZHD initiated and led the research, overseeing data collection and contributing to all aspects of manuscript development and finalization. HD and EH were involved in data analysis, literature review, and manuscript development from the initial phases to the finalization of the manuscript. All authors approved the submitted version.

## Conflict of interest

The authors declare that the research was conducted in the absence of any commercial or financial relationships that could be construed as a potential conflict of interest.

## Publisher’s note

All claims expressed in this article are solely those of the authors and do not necessarily represent those of their affiliated organizations, or those of the publisher, the editors and the reviewers. Any product that may be evaluated in this article, or claim that may be made by its manufacturer, is not guaranteed or endorsed by the publisher.
